# Investigation of Aerogel Production Processes: Solvent Exchange under High Pressure Combined with Supercritical Drying in One Apparatus

**DOI:** 10.3390/gels7010004

**Published:** 2021-01-05

**Authors:** Artem Lebedev, Ekaterina Suslova, Aleksander Troyankin, Daria Lovskaya

**Affiliations:** International Center for Transfer of Pharmaceutical and Biotechnology, Mendeleev University of Chemical Technology of Russia, 125047 Moscow, Russia; artem.evg.lebedev@gmail.com (A.L.); suslova.ekaterina.nik@mail.ru (E.S.); a.troyankin@gmail.com (A.T.)

**Keywords:** aerogel, solvent exchange, ternary phase equilibria, supercritical fluids, intensification

## Abstract

This work aims to contribute to the theoretical and experimental research of supercritical processes for intensification and combination in one apparatus. Investigation is carried out to improve production technology of organic alginate aerogels. It is proposed within the investigation to carry out the solvent exchange stage, an important stage of organic aerogels production, under pressure in a carbon dioxide medium in the same apparatus used for supercritical drying. The phase behavior in the system “carbon dioxide–water–2-propanol”, which arises during such a solvent exchange stage, is studied theoretically. An experimental study of the process of step-by-step solvent exchange under pressure was carried out through multiphase and homogeneous regions of the phase diagram of such a system. As a result, new highly efficient technology for the production of organic aerogels was proposed, which can be implemented by combining the two main stages of the process.

## 1. Introduction

A supercritical fluid has the properties of gases, such as high diffusion rate, low viscosity and compressibility, as well as the following properties of liquids: high density and high dissolving power. These properties define the high intensity of mass transfer in the medium of the supercritical fluid. Supercritical fluids are widely used in processes of extraction [1], micronization [2], chromatography [3] and impregnation [4]. Furthermore, supercritical fluids are used to obtain aerogel [5,6,7]—an innovative material with properties such as low density, high porosity and specific surface area. Aerogels can be prepared using various initial materials: organic [8,9], inorganic [10,11,12] and their mixture. Due to these properties, aerogels have a number of promising applications [12,13,14,15]: from thermal insulation, sensors and catalysts to drug-delivery systems, tissue engineering and hemostatic agents.

The process of obtaining organic aerogels includes three stages: gelation, solvent exchange and supercritical drying. To form organic gels, various polysaccharides (cellulose, starch, sodium alginate, pectin, chitosan, etc.), proteins (egg, soy, etc.), polymers (polypyrrole, polydimethylsiloxane, etc.) and resins (phenol-formaldehyde, resorcinol-formaldehyde, etc.) can be used. For organic components, gelation can be initiated by both chemical (e.g., the use of a crosslinking agent [16,17]) and physical factors (e.g., changes in temperature or pH of the medium [18]). The formation of organic gels takes place in an aqueous medium. Since water is immiscible with supercritical carbon dioxide, before the drying process it is necessary to replace water inside the gel structure with a solvent which forms a homogeneous system with supercritical carbon dioxide. The most commonly used organic solvents are methanol, ethanol, 2-propanol and acetone. The solvent exchange stage is carried out stepwise [19,20] since organic solvents and water have different surface tensions and pore collapse may occur because of concentration gradient. This collapse will lead to the destruction of gel structure and shrinkage of material. Therefore, gel samples are transferred from water to solutions of water and an organic solvent with a sequential increase in the concentration of the organic solvent. At the final steps of solvent exchange, the gels are placed several times in a pure organic solvent. In total, a significant time from tens of hours to several days is spent on the solvent exchange. After completion, the gels are placed in a high-pressure apparatus, and the supercritical drying process is carried out.

To increase the efficiency of the production process of the organic gels the stages of solvent exchange and supercritical drying can be combined in one apparatus. The effectiveness of such a combination has been shown for other supercritical processes [21]. The solvent exchange can be carried out in a supercritical dryer, whereby the water can be changed to an organic solvent in the presence of pressurized carbon dioxide. Under such conditions, the mass transfer of the solvent from the pores of the gel proceeds more intensively than at atmospheric pressure, which leads to a significant reduction in the time of the solvent exchange stage. After the completion of such a solvent exchange in the same apparatus, supercritical drying can be carried out in the same way. This method makes it possible to significantly reduce the number of operations for loading and unloading equipment, to reduce the cost of transporting intermediate products and, in general, to reduce the cost of producing organic aerogels.

During the stage of the solvent exchange under pressure, a three-component system “carbon dioxide–water–organic solvent” is formed. Depending on the composition, this system can form multiphase regions. When considering multicomponent systems under pressure, it is necessary to calculate the following basic physicochemical quantities: density, viscosity and the molecular diffusion coefficient. When calculating the density, empirical equations [22,23,24,25] can be used, which differ in complexity, and each of them can be selected depending on the required calculation accuracy. In addition, the most commonly used method for determining the density in such multicomponent systems is the use of cubic equations of state together with mixing rules. Table 1 lists the various equations of state, and Table 2 lists the mixing rules.

A number of different equations can be used to calculate the dynamic viscosity of the systems under examination [23,25,26,27,28,29]. The equation in [27] is most accurate. To take into account the effect of the composition of the system on its dynamic viscosity, linear or logarithmic dependences can be used. As the analysis of literary sources has shown, the use of simpler, linear dependences often allows results with sufficient accuracy to be obtained and significantly reduces the complexity of the calculation. This is especially true for conditions when the temperature and pressure are above critical levels. There are a number of empirical and semi-empirical equations for calculating the molecular diffusion coefficient [23,30,31,32,33,34,35,36]. 

Thus, there are many equations for the calculation of the physicochemical properties of multicomponent systems under pressure in a carbon dioxide medium. Within the framework of this work, the Peng–Robinson equation will be used together with the van der Waals mixing rule for the state calculation of a three-component system.

Within the framework of this work the ternary system “carbon dioxide–water–2-propanol” was theoretically studied. On the obtained basis the parameters of the solvent exchange process under pressure in the medium of carbon dioxide were selected. An experimental study of the process of the solvent exchange under pressure was carried out using two approaches: (1) operating line of the process passes through the region of existence of a multiphase system, (2) operating line of the process passes only through the region of existence of a homogeneous system. Finally, a method for calculation of operating lines of the process was proposed and the most efficient solvent exchange process under pressure in a carbon dioxide medium was determined.

## 2. Results and Discussion

### 2.1. Theoretical Investigation of ‘Carbon Dioxide–Water–2-Propanol’ Ternary Sistem

The use of carbon dioxide at the stage of the solvent exchange leads to consideration of the three-component system “carbon dioxide–water–2-propanol”. Consideration of a three-component system requires the study of phase equilibrium for various external parameters. According to the Gibbs phase rule, such a system can form a maximum of five phases. In the presence of five phases the system has no degrees of freedom, it is invariant, which is possible only with strictly defined parameters (temperature, pressure, composition) and their change will cause changing phase equilibrium and a decreasing number of phases. During the process of solvent exchange, the composition of the system is constantly changing, therefore, at fixed pressure and temperature, the three-component system have only two degrees of freedom in composition. Therefore, in such a system, during the entire solvent exchange process, up to three different phases can coexist simultaneously.

**Figure 1:**
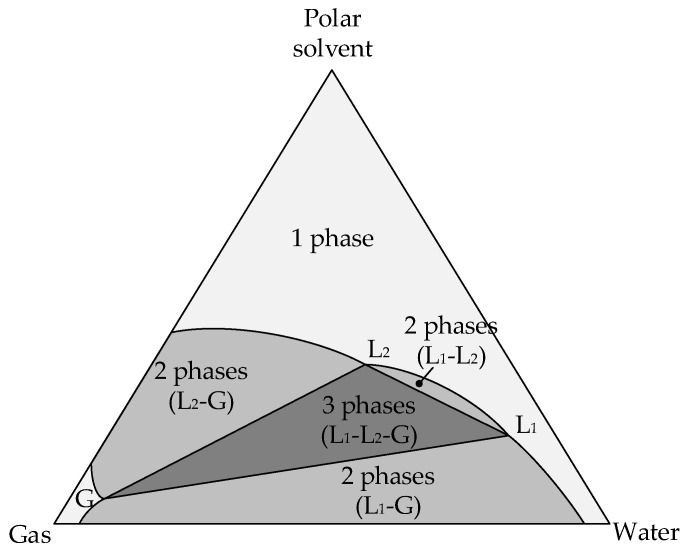
Typical phase diagram for “gas–water–polar solvent” systems at high pressure [43].

Figure 1 shows a typical phase diagram for “gas–water–polar solvent” system at high pressure, where L_1_ is the liquid phase with a higher water content, L_2_ is the liquid phase with a higher content of polar solvent (2-propanol for current research), G is the gas phase with higher content of carbon dioxide.

The diagram shows the regions of composition of the system at which different phase equilibrium occurs. The area in which there are three phases is bounded by a triangle, its vertices (L_1_, L_2_, G) correspond to the composition of these three phases. When the system forms two phases, their composition can be determined by the conodes-lines connecting the compositions of the two equilibrium phases. The phase equilibrium of the considered system “carbon dioxide–water–2-propanol” changes significantly with increasing pressure [43]. Under various conditions, two-phase and three-phase regions can form in this system. If we consider the specified system at a temperature of 40 °C and when the pressure changes from 78 to 98 bar, then important critical points can be found: a pressure of 78.1 bar, at which the first region of existence of three phases appears, a pressure of 78.5 bar, at which a second region of existence of three phases appears, and a pressure of 97.1 bar, at which the regions of existence of three phases disappear.

In the framework of the previous preliminary work [44], it was first proposed to carry out the solvent exchange stage under pressure in the same apparatus as supercritical drying. The behavior of the system was studied during one step of solvent exchange under pressure in a carbon dioxide medium from 0% to 50% 2-propanol concentration at a temperature of 40 °C. The process was carried out at pressures of 80 and 100 bar so that the operating line of the process on the phase diagram of the “carbon dioxide–water–2-propanol” system passes through the region of existence of three phases and the region of existence of two phases. It was shown that with the occurrence of a larger number of phases, the process proceeds more intensively. In addition, it was concluded that this method of solvent exchange can significantly reduce the process time and consumption of the organic solvent in comparison with traditional solvent exchange without pressure.

Thus, in the current study, solvent exchange is being investigated at pressure of 80 bar pressure and temperature of 40 °C.

### 2.2. Experimental Investigation of the Solvent Exchange Process under High Pressure

**Figure 2:**
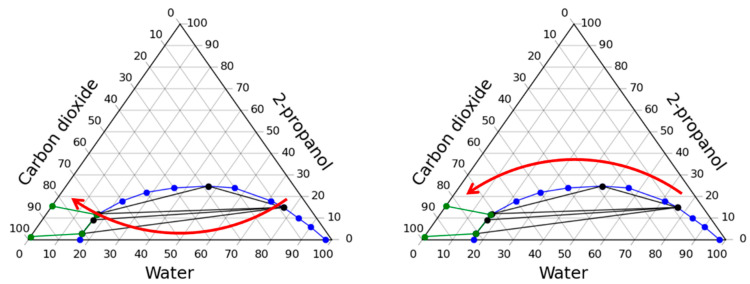
Two approaches to carrying out the process of the solvent exchange under pressure in a carbon dioxide medium: through the multiphase region (**left**) and through the homogeneous region (**right**).

The experimental study of the solvent exchange process was carried out using two main approaches. In the first approach, solvent exchange steps are chosen so that the system inside the apparatus is predominantly multiphase, and in the second, it is homogeneous. Figure 2 shows the phase diagram of the “carbon dioxide–water–2-propanol” system which demonstrates the courses of operating lines of the process when using the above approaches.

All experiments were carried out at pressure in the apparatus up to 80 bar and at temperature 40 °C. The loading of the gel into the apparatus in all cases was 100 mL, in addition, together with the gel, 30 g of water was added into the apparatus. Thus, it is assumed that the total mass of water inside the apparatus is 129 g. The average particle diameter of the gel that is loaded into the apparatus was 2.7 mm with standard deviation equal to 0.14.

In the case of successful solvent exchange, supercritical drying was performed. It was considered successful if, upon completion, there was no visible shrinkage of gel particles visible through the sight glasses of the high-pressure apparatus. The influence of various variants of the process on the quality of the material was investigated. 

#### 2.2.1. Solvent Exchange through Multiphase Region of Phase Diagram 

When carrying out the process through the multiphase region of the phase diagram, the following parameters were varied:

- experiments 1 and 2—the composition was changed to follow mass concentration of 2-propanol sequence 10–30–50–70–90–100–100 wt.%; carbon dioxide feed was started at a step when mass concentration of alcohol was 10% to achieve a pressure of 80 bar;
- experiments 3 and 4—the composition was changed to follow mass concentration of 2-propanol sequence 10–30–50–60–70–80–90–100 wt.%; carbon dioxide feed was started at a step when mass concentration of alcohol was 10% to achieve a pressure of 80 bar;
- experiments 5 and 6—the composition was changed to follow mass concentration of 2-propanol sequence 10–30–50–60–70–80–90–100–100 wt.%; carbon dioxide feed was started at a step when mass concentration of alcohol was 10% to achieve a pressure of 80 bar;
- experiments 7—the composition was changed to follow mass concentration of 2-propanol sequence 10–30–50–70–80–90–100–100–100 wt.%; carbon dioxide feed was started at a step when mass concentration of alcohol was 50% to achieve a pressure of 80 bar;
- experiments 8—the composition was changed to follow mass concentration of 2-propanol sequence 10–30–50–60–70–90–100–100 wt.%; carbon dioxide feed was started at a step when mass concentration of alcohol was 10% to achieve a pressure of 80 bar.

**Figure 3:**
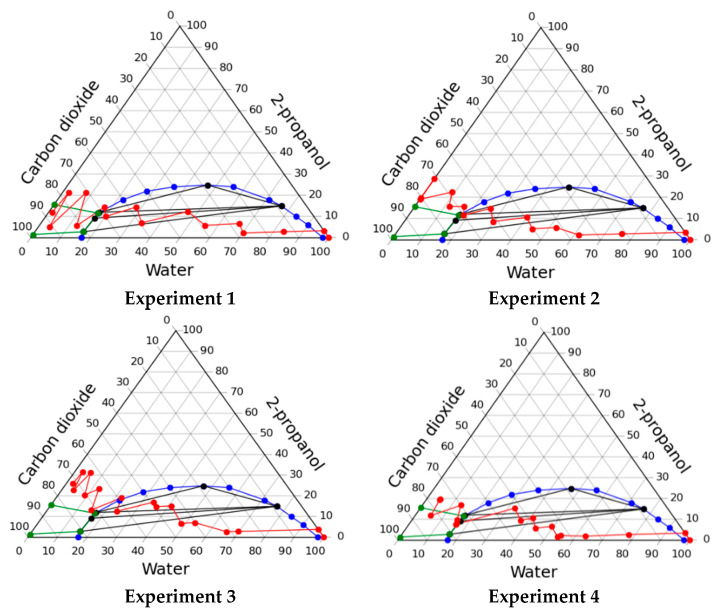

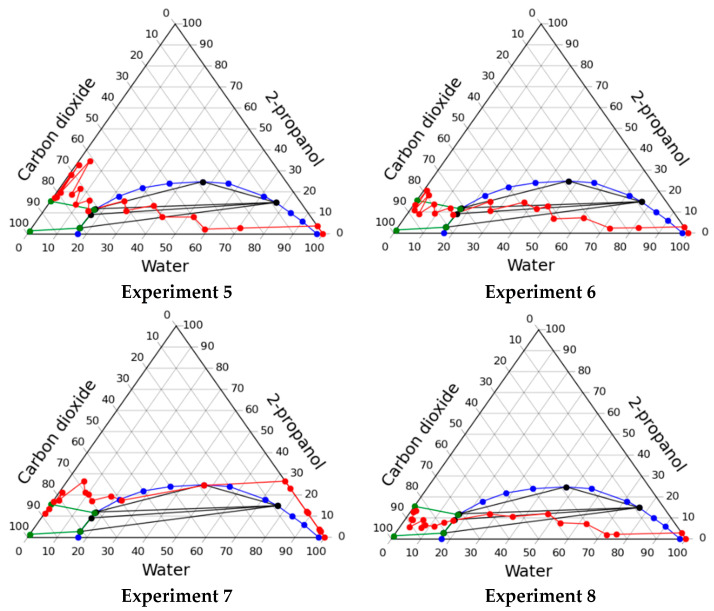
Operating lines of the solvent exchange process through multiphase region in various experiments.

The resulting operating lines of the solvent exchange process are presented in Figure 3, and Table 3 shows the consumption of organic solvent and carbon dioxide, as well as the shrinkage of the material.

In the course of this experiment, significant shrinkage (>50%) of gels occurred. This shrinkage is associated with the appearance of a phase boundary inside the gel, which causes collapse of pores due to capillary forces. A significant shrinkage of the material shows its unsatisfactory quality. Despite the unsatisfactory quality it can be supposed that carrying out the process in a multiphase region is highly promising. But to implement such a regime, it is necessary to maintain such conditions inside the apparatus so that the phase boundary does not form within the porous body (gel). This requires solving the problem of high-level automation and control of process parameters and demands significant costs.

#### 2.2.2. Solvent Exchange through Homogeneous Region of Phase Diagram 

Furthermore, a series of experiments were carried out in a homogeneous region without alginate gel to test the behavior of the system under various conditions. Such studies are necessary in order to identify the required number of steps and the necessary sequence of pressure increase to ensure that the system is located only in the homogeneous region of the phase diagram. In each experiment, 129 g of water was loaded into the apparatus, which corresponds to the mass of water in previous experiments. Parameters of experiments are presented below:

- experiment 9—the composition was changed to follow mass concentration of 2-propanol sequence 10–30–50–60–70–80–90–100–100 wt.%; carbon dioxide feed was started at a step when mass concentration of alcohol was 50%; at each step starting from a step of 50%, the pressure in the apparatus increased as follows: 10–20–30–40–50 bar;
- experiment 10—the composition was changed to follow mass concentration of 2-propanol sequence 10–30–50–60–70–80–90–100–100 wt.%; carbon dioxide feed was started at a step when mass concentration of alcohol was 50%; at each step starting from a step of 50%, the pressure in the apparatus increased as follows: 10–20–30–80–80 bar;
- experiment 11—the composition was changed to follow mass concentration of 2-propanol sequence 10–30–50–60–70–80–90–100–100 wt.%; carbon dioxide feed was started at a step when mass concentration of alcohol was 50%; at each step starting from a step of 50%, the pressure in the apparatus increased as follows: 10–20–50–60–90 bar.

**Figure 4:**
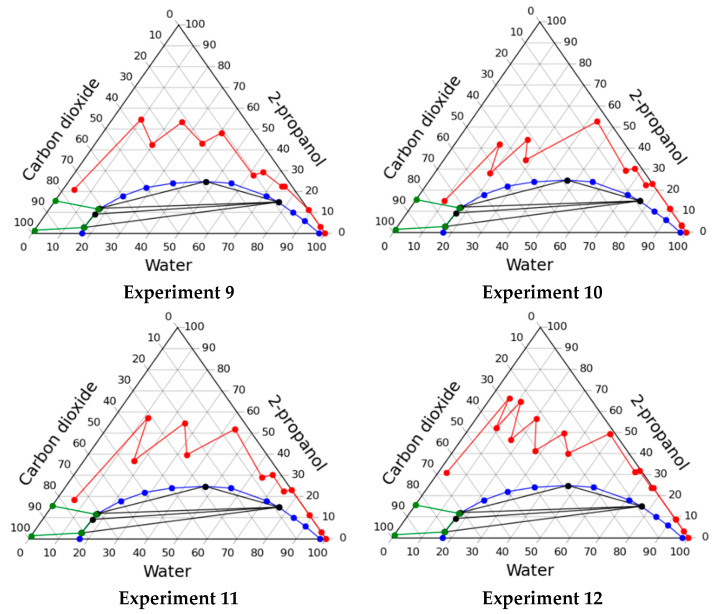
Operating lines of the solvent exchange process through homogeneous region in various experiments.

The data obtained, namely, operating lines of the process on the phase diagram, the consumption of alcohol and carbon dioxide are presented in Figure 4 and in Table 4.

It can be seen from the data that the lowest consumption of 2-propanol can be obtained when carrying out the process of the solvent exchange in accordance with the regime of experiment 10. It is important to note that in all cases the system was in a homogeneous state, it was confirmed visually during the experiments. 

Solvent exchange processes through homogeneous region with alginate gel were carried out according to the regime of experiment 10:

- experiment 12—the composition was changed to follow mass concentration of 2-propanol sequence 10–30–50–60–80–90–95–100–100 wt.%; carbon dioxide feed was started at a step when mass concentration of alcohol was 50%; at each step starting from a step of 50%, the pressure in the apparatus increased as follows: 10–20–30–80–80–80 bar;

It should be noted that in this case an additional step of solvent exchange was added to ensure that the least amount of water inside the gel is reached. The data obtained are also presented in Figure 4 and Table 4. The particles obtained after solvent exchange were supercritically dried at pressure of 120 bar temperature of 40 °C and a flow rate of carbon dioxide 500 g/h. The resulting aerogels properties shown in the Table 5. Properties of the aerogel particles produced by the dripping method from other research are also shown in the Table. As can be seen from the data, combination of solvent exchange and supercritical drying in one apparatus allows to produce particles of alginate aerogel with comparable properties. 

As can be seen from the data alginate aerogel quality is similar to the results from other works. This confirms the possibility to use the solvent exchange approach through the single-phase region of the phase diagram of the system.

Based on the results obtained, it can be concluded that carrying out the stage of solvent exchange through the multiphase region of the phase diagram can significantly reduce the consumption of alcohol and carbon dioxide. But this approach does not allow a material to be produced with appropriate quality. Based on the results of experimental studies, it is proposed to use the solvent exchange approach through the single-phase region of the phase diagram of the system. This is due to the fact that it allows material to be obtained with desired properties.

### 2.3. Intensification of the Solvent Exchange Process under High Pressure 

In order to reduce the consumption of solvents for the implementation of the solvent exchange stage under pressure, the process was intensified using the proposed algorithm for calculating the theoretical operating lines of the stage (Section 4.5). This algorithm allows the number of solvent exchange steps and the target composition of the system inside the apparatus to vary, and the total consumption of 2-propanol and carbon dioxide, and the resulting pressure at each step of solvent replacement in a semi-automatic mode to be calculated.

**Figure 5:**
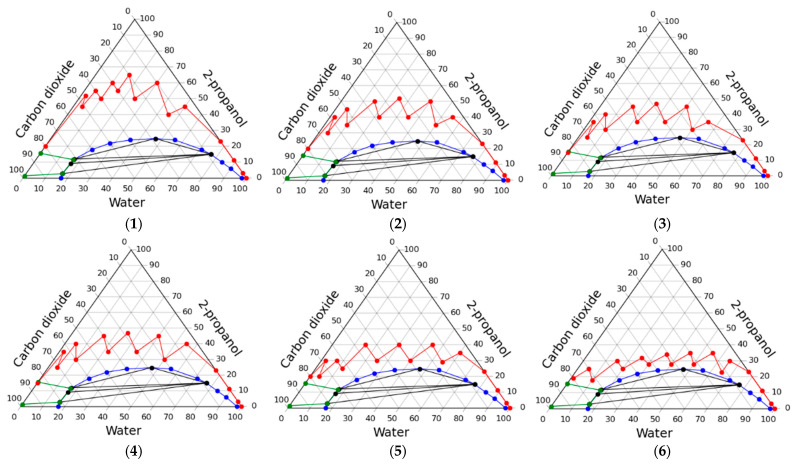
Intensification of the solvent exchange under high pressure at carbon dioxide medium: operating line examples.

Using the algorithm, a series of working lines of the solvent exchange process were obtained, some of which are shown in Figure 5. The aim of intensification was to find the minimum consumption of 2-propanol, with condition that the process is carried out in a single-phase. The figure shows the resulting reduction in these costs. Quantitative values are presented in Table 6.

Thus, from the obtained data, it can be concluded that the solvent exchange in a single-phase system with the lowest solvents consumption is possible when the operating line of the process is as close as possible to the phase separation line in the phase diagram. It is relevant to find a method for the solvent exchange procedure not in step-by-step mode, but in the mode of smooth concentration change. This is possible if a continuous supply of 2-propanol, carbon dioxide and a continuous discharge of the resulting mixture are organized in the apparatus. Such an organization of the process is highly complex but, especially at the industrial level, this will significantly reduce costs.

## 3. Conclusions

Within the framework of the study, the phase behavior of the multicomponent system “carbon dioxide–water–2-propanol”, which arises during the solvent exchange under pressure in the medium of carbon dioxide, was theoretically shown. The information obtained was used to select the parameters of carrying out this process.

The process of the solvent exchange was experimentally investigated using two approaches: carrying out the process through a multiphase and through a homogeneous region of the phase diagram of the “carbon dioxide–water–2-propanol” system. It was shown that the exchange through the multiphase region is more efficient, with less raw material consumption, but it is not possible to obtain aerogel of satisfactory quality. At the same time, solvent exchange through the homogeneous region, while having high raw material consumption, allows the required material quality to be obtained. The gel obtained after such exchange was successfully dried supercritically. As a result, the aerogel particles with comparable quality were produced.

The necessary dependences of the process of solvent exchange under pressure in a carbon dioxide medium on the parameters were established. An algorithm that allow a quick calculation of the process of solvent exchange under pressure was developed.

A new highly efficient technology for the production of aerogels has been proposed. It is implemented by combining two main stages of the process, solvent exchange and supercritical drying, in one apparatus. Implementation of this technology will significantly reduce the capital costs of organizing the industrial production of aerogels by carrying out several stages of the process in one apparatus. In addition, operating costs could be reduced too by decreasing the consumption of organic solvents.

## 4. Materials and Methods

### 4.1. Chemicals

Alginic acid sodium salt (AlgNa, Algin, Alginic acid sodium salt from brown algae) (CAS 9005-38-3), Sigma-Aldrich, Saint Louis, MO, USA; CaCl_2_·2H_2_O (CAS 10035-04-8) purity ≥ 99.5%, Sigma-Aldrich; 2-propanol, purity ≥ 99.5%, RusHim, Moscow, Russia. All chemicals were used directly without further purification.

### 4.2. Preparation of Sodium Alginate Gels

**Figure 6:**
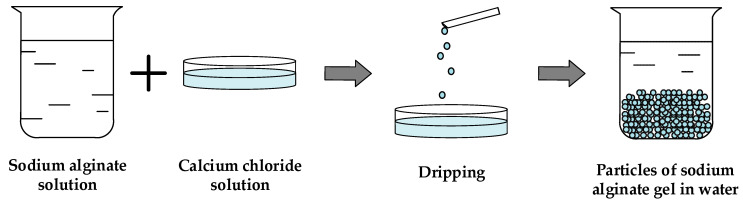
Schematic diagram of sodium alginate gel particles obtainment trough dripping procedure.

To obtain spherical particles of sodium alginate gel dripping method was used. The detailed description of the method presented in [48]. Within the framework of this method, a 1 wt.% solution of sodium alginate and 5 wt.% solution of calcium chloride in water were prepared. Then a solution of sodium alginate is dripped into the solution with a crosslinking agent (calcium chloride) through a needle with constant stirring. The resulting gel particles are kept in a CaCl_2_ solution for a 24 h while the ongoing chemical reactions take place. Schematic diagram of sodium alginate gel particles obtainment is shown in Figure 6.

### 4.3. Method of the Investigation of Solvent Exchange under High Pressure 

**Figure 7:**
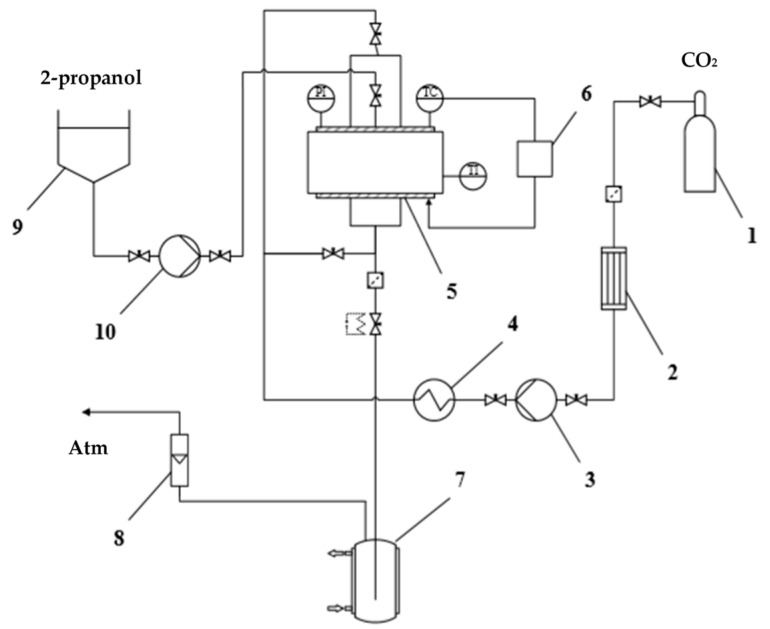
Schematic diagram of the setup for supercritical processes.

The study of solvent exchange under pressure was carried out using equipment of our own design for carrying out supercritical processes [49,50]. This equipment has been further upgraded to enable the supply of an organic solvent along with carbon dioxide under pressure. The schematic diagram of the setup used is shown in the Figure 7. The setup consists of a carbon dioxide supply line, a 250 mL high-pressure apparatus 5, which is equipped with sight glasses, pressure gauge PI, temperature sensor TC, and temperature controller 6, a medium discharge line, and an organic solvent supply line, which is added additionally. The carbon dioxide supply line consists of carbon dioxide tank 1, a condenser 2, a liquid pump 3 (Maximator, G35), a heating element 4. The medium discharge line consists of a separator 7 for collecting the liquid phase, a rotameter 8 for measuring the flow rate of the gas phase and a pipeline that connects the system with the atmosphere. The organic solvent supply line consists of a dosing container 9, a membrane dosing pump 10 (Lewa, LDB1), a pipeline and valves. This line, as well as the carbon dioxide supply line, is connected to one of the upper branch pipes of the apparatus. The setup includes a Coriolis flow meter (Bronkhorst, mini Cori-flow M13) on the carbon dioxide supply line, which monitors the mass flow rate of the gas and determines its total mass. 

When investigating solvent exchange, the gel is loaded into the apparatus then, in accordance with the selected sequence, 2-propanol and/or carbon dioxide are fed into the apparatus and the preset process parameters are set. The volume of 2-propanol supplied to the apparatus is controlled by the liquid level in the dosing container 9 with an accuracy of 0.1 mL. Its flowrate is maintained at 50–100 mL/min and the process takes a few minutes. The supply of carbon dioxide to the apparatus is controlled according to Coriolis flow meter, so that the flowrate of carbon dioxide does not exceed 1000 g/h. It is necessary to prevent destruction and shrinkage of final material structure. The carbon dioxide flowrate is regulated using a pressure reducer. Processing of flow meter data allows the actual mass of carbon dioxide supplied to the apparatus at each step of solvent exchange process to be determined. After reaching the specified parameters at each step of solvent exchange the system is kept for 20 min. Then, part of the medium from the apparatus is drained into the separator. The 2-propanol content in the drained medium is determined spectrophotometrically.

Thus, each step of the solvent exchange process consists of the following substeps:

- Supply of a given amount of 2-propanol to reach the desired concentration according to stepwise solvent exchange procedure;
- Supply of the required amount of carbon dioxide to achieve or maintain the specified pressure;
- Draining part of “carbon dioxide–water–2-propanol” medium from the apparatus.

The achievement of specified pressure takes place at a step when carbon dioxide feed is started. The maintaining of specified pressure takes place together with draining. The certain amount of 2-propanol at each step of the process is calculated based on desired stepwise exchange procedure. The required mass of 2-propanol on the current step is determined based on the target concentration of “water–2-propanol” mixture and the masses of these components at the end of the previous step after draining.

### 4.4. Analytical Methods

To determine the shrinkage of material, the diameter of 20 gel particles was measured immediately before the beginning of solvent exchange and after supercritical drying. Mean particle diameter and its standard deviation were calculated. The shrinkage was calculated as the relative change in the diameter of the aerogel particles in comparison with the diameter of the gel before the beginning of the solvent exchange. If supercritical drying was not carried out, when significant shrinkage was observed after the end of the solvent exchange, the shrinkage was determined by the diameter of the gel particles after the solvent exchange.

Bulk density of alginate aerogel particles was determined by dividing average mass of particle to the average particle volume. Average mass of particle was obtained by weighing more than 20 particles using a balance. Particle volume was calculated using average particle diameter.

The textural characterization of the aerogels was carried out by low-temperature N_2_ adsorption—desorption analysis (ASAP 2020MP, Micromeritics, Norcross, GA, USA). Prior to measurements, samples were dried under a vacuum (<1 MPa) at 60 °C for 20 h. Specific surface area was determined by the BET (Brunauer–Emmett–Teller) method. BJH (Barrett–Joyner–Halenda) analysis was employed to determine average pore diameter using desorption techniques.

### 4.5. Calculation of the Solvent Exchange Operating Line

The article proposes two algorithms:

- algorithm for processing experimental data, which allows to build experimental operating lines of the process on phase diagrams;
- algorithm for calculating the theoretical operating lines of the solvent exchange process and plotting them on phase diagrams.

**Figure 8:**
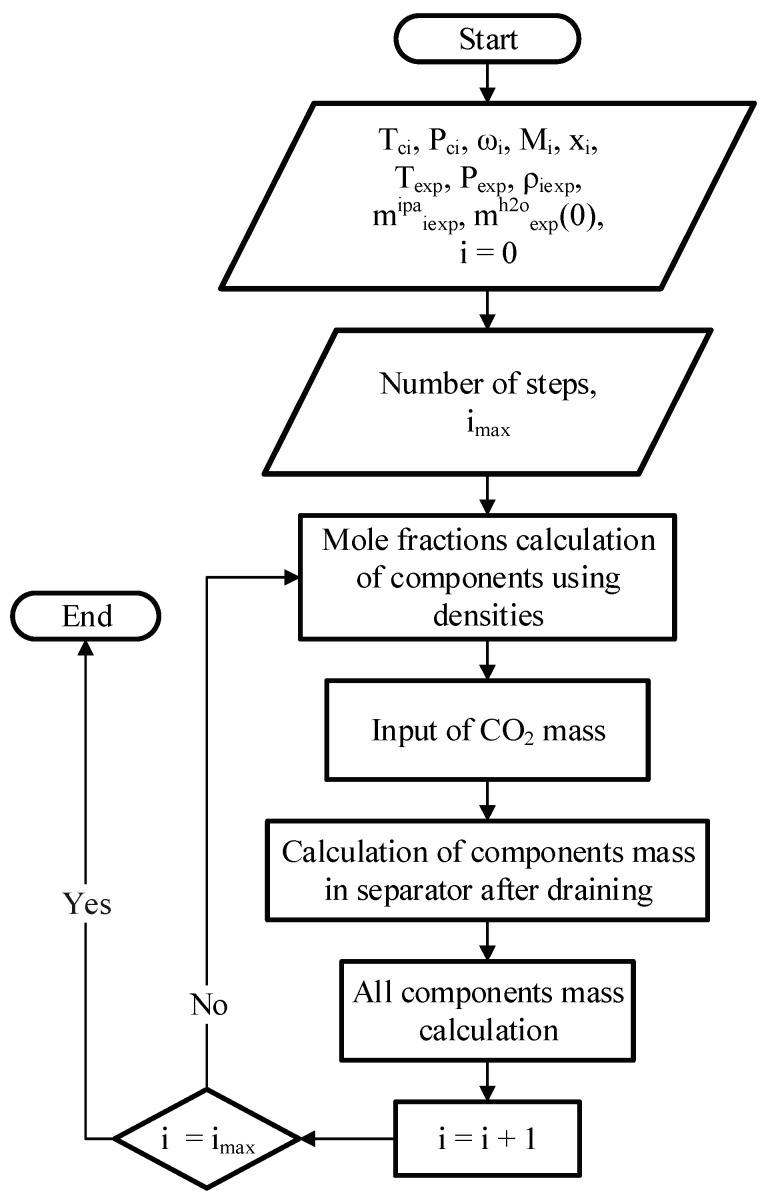
Algorithm for processing experimental data.

The algorithm for processing experimental data is shown in Figure 8.

Initial data for calculation: parameters of substances (critical temperature, critical pressure, acentric factor and molar mass); operating temperature and pressure; the density of the mixtures in separator after draining at the end of each step of solvent exchange; masses of 2-propanol and carbon dioxide added at each step of solvent exchange; the mass of water at the zero step; the number of steps of solvent exchange. The composition of the system at each step of solvent exchange was determined based on the mass and composition of the system at the previous step, the mass of 2-propanol and carbon dioxide added, and the mass and composition of the drain from the apparatus at the next step. Based on the data on the molar concentrations of substances obtained at each step, the operating line of the process was built on the phase diagram of the three-component system “carbon dioxide–water–2-propanol”.

The algorithm for calculating the theoretical operating lines of the solvent exchange process is based on the algorithm for processing experimental data. At the same time, it does not use experimental values as initial data, but sets the required number of steps, and sets the limits of possible changes in concentration at each of the steps based on the researcher’s experience. Further, within the framework of the algorithm, the composition of the system is iteratively varied at each step so that such a composition is attainable. As a result, a number of different operating lines of the solvent exchange process can be obtained. For each of these lines, the program calculates the consumption of 2-propanol and carbon dioxide.

The proposed algorithms were implemented using the Python 3.8, Python Software Foundation, Wilmington, DE, USA.

## Figures and Tables

**Table 1:** List of equations of state suitable for calculating the thermodynamic characteristics of substances under sub- and supercritical conditions.

No.	Name	Equations	Description	Reference
1	Modified Redlich–Kwong for carbon dioxide	$P=\frac{R\cdot T}{V-b}-\frac{\beta \cdot a}{V\cdot \left(V+b\right)},$ $a=0.42748\cdot \frac{{\left(R\cdot T_{c}\right)}^{2}}{P_{c}},$ $b=0.0866\cdot \frac{R\cdot T_{c}}{P_{c}},$ $\beta =\frac{\beta_{1}+\beta_{2}\cdot \ln\left(T_{r}\right)+\beta_{3}\cdot \ln\left(P_{r}\right)}{1+\beta_{4}\cdot \ln\left(T_{r}\right)+\beta_{5}\cdot \ln\left(P_{r}\right)+\beta_{6}\cdot {\left[\ln\left(P_{r}\right)\right]}^{2}}$	At temperatures from 305 to 1100 K and pressures from 7.645 to 800 MPa, the deviation of the calculated data is relatively small (13%)	[37]
2	Soave–Redlich–Kwong for non-polar substances	$P=\frac{R\cdot T}{V-b}-\frac{a}{V\cdot \left(V+b\right)},$ $a={\left[1+m\cdot \left(1-\sqrt{\frac{T}{T_{c}}}\right)\right]}^{2},$ $m=0.480\cdot \omega +1.574\cdot \omega -0.176\cdot \omega^{2}$	At pressures from 3 to 12 MPa, the average deviation of the calculated data from the experimental is 4.4%	[38]
3	Peng–Robinson equation	$P=\frac{R\cdot T}{V-b}-\frac{a}{V\cdot \left(V+b\right)+b\cdot \left(V-b\right)},$ $a=0.4572\cdot \frac{{\left(R\cdot T_{c}\right)}^{2}}{P_{c}}\cdot {\left[1+m\cdot \left(1-\sqrt{\frac{T}{T_{c}}}\right)\right]}^{2},$ $b=0.0778\cdot \frac{R\cdot T_{c}}{P_{c}},$ $m=0.3746+1.54226\cdot \omega -0.2699\cdot \omega^{2}$	In the near-critical temperature range, the error is 4.4%	[39]
4	Patel–Teja equation	$P=\frac{R\cdot T}{V-b}-\frac{a}{V\cdot \left(V+b\right)+c\cdot \left(V-b\right)},$ $a=\Omega_{a}\cdot \frac{{\left(R\cdot T_{c}\right)}^{2}}{P_{c}}\cdot a\left(T_{r}\right),$ $b=\Omega_{b}\cdot \frac{R\cdot T_{c}}{P_{c}},\;c=\Omega_{c}\cdot \frac{R\cdot T_{c}}{P_{c}},$ $\Omega_{a}=3\cdot \zeta_{c}{}^{2}+3\cdot \left(1-2\cdot \zeta_{c}\right)\cdot \Omega_{b}+\Omega_{b}{}^{2}+1-3\cdot \zeta_{c},$ $\Omega_{c}=1-3\cdot \zeta_{c},$ $\zeta_{c}=\frac{P_{c}\cdot V_{c}}{R\cdot T_{c}},$ $a\left(T_{пp}\right)={\left[1+\omega \cdot \left(1-T_{пp}{}^{1/2}\right)\right]}^{2},$ $\Omega_{b}$—smallest positive root of an equation: $\Omega_{b}{}^{3}+\left(2-3\cdot \zeta_{c}\right)\cdot \Omega_{b}{}^{2}+3\cdot \zeta_{c}{}^{2}\cdot \Omega_{b}-\zeta_{c}{}^{3}=0$	In the near-critical region of temperatures and pressures, the average deviation of the calculated data from the experimental is 4.48%	[40]
5	Equation of state for near-critical and critical regions	$\frac{P}{R\cdot T}=\frac{1}{V}+\sum_{n=2}^{6}\frac{a_{n}}{V^{n}}+\sum_{m=1}^{2}\frac{a_{m+6}}{1+e_{m}\cdot {\left(V-V_{c}\right)}^{2}}+\sum_{k=1}^{2}a_{k+8}\cdot \exp\left(-\alpha_{k}\cdot V\right),$ $a_{i}=\sum_{j=0}^{J_{i}}a_{ij}\cdot \theta^{j},\;\left(i=2-10\right),$ $\theta =T_{r}{}^{-1}-1$	At temperatures from 304 to 647 K and pressures up to 80 MPa, the deviation of the calculated data from the experimental was less than 1%	[41]
6	Modified Lee–Kesler equation	$Z=1+\frac{B}{V_{r}}+\frac{C}{V_{r}{}^{2}}+\frac{D}{V_{r}{}^{4}}+\frac{E}{V_{r}{}^{5}}+\frac{F}{V_{r}{}^{2}}\cdot \left(\beta +\frac{\gamma }{V_{r}{}^{2}}\right)\cdot \exp\left(-\frac{\gamma }{V_{r}{}^{2}}\right),$ $B=a_{1}+\frac{a_{2}}{T_{r}{}^{2}}+\frac{a_{3}}{T_{r}{}^{3}},$ $C=a_{4}+\frac{a_{5}}{T_{r}{}^{2}}+\frac{a_{6}}{T_{r}{}^{3}},$ $D=a_{7}+\frac{a_{8}}{T_{r}{}^{2}}+\frac{a_{9}}{T_{r}{}^{3}},$ $E=a_{10}+\frac{a_{11}}{T_{r}{}^{2}}+\frac{a_{12}}{T_{r}{}^{3}},$ $F=\frac{\alpha }{T_{r}{}^{3}}.$	At temperatures from 273 to 1273 K and pressures up to 350 MPa, the deviation of the calculated data from the experimental is insignificant (up to 0.5%)	[42]

**Table 2:** List of mixing rules [23].

No.	Name	Equations	Description
1	Van der Waals mixing rule	$a_{mix}=\sum_{i=1}^{n}\sum_{j=1}^{n}y_{i}y_{j}a_{ij}$ $b_{mix}=\sum_{i=1}^{n}y_{i}b_{ii}$ $a_{ij}=\left(1-k_{ij}\right){\left(a_{ii}a_{jj}\right)}^{0.5}$ $k_{ij}$—empirical binary interaction parameter	Used for a wide range of substances
2	Mixing rule	$a_{mix}=\sum_{i=1}^{n}\sum_{j=1}^{n}y_{i}y_{j}a_{ij}+f_{NC}$ $f_{NC}=\sum_{i=1}^{n}\sum_{j=1}^{n}y_{i}y_{j}a_{ij}(k_{ij}x_{i}+k_{ji}x_{j})$ $a_{ij}={\left(a_{ii}a_{jj}\right)}^{0.5}$ $b_{mix}=\sum_{i=1}^{n}y_{i}b_{ii}$	This type of equations is used to calculate when mixing non-polar substances with polar or associated components.
3	Huron–Vidal mixing rule	$a_{mix}=b_{mix}\left[\sum_{i=1}^{n}\frac{x_{i}a_{i}}{b_{i}}+\frac{G^{E}}{C\left(V\right)}\right]$ $G^{E}$—excess free Gibbs energy The parameter C (V) depends on the chosen equation of state, for example, for the Peng-Robinson equation of state it is –0.6232 $b_{mix}=\sum_{i=1}^{n}y_{i}b_{ii}$	Mixing rules based on the calculation of the thermodynamic potentials of the system

**Table 3:** Consumption of components and gel shrinkage during the solvent exchange process through multiphase region.

No.	2-Propanol Consumption, g	CO_2_ Consumption, g	Gel Shrinkage, %
1	261	764	55.4
2	237	581	57.3
3	387	659	53.8
4	250	647	74.9
5	398	767	56.5
6	336	947	58.2
7	288	794	60.2
8	287	964	65.0

**Table 4:** Consumption of components and gel shrinkage during the solvent exchange process through homogeneous region.

No.	2-Propanol Consumption, g	CO_2_ Consumption, g	Gel Shrinkage, %
9	275	62	-
10	268	241	-
11	642	341	-
12	414	441	5.9

**Table 5:** Alginate aerogel properties in this study and in other research.

No.	ρ_bulk_	Surface Area m^2^/g	Average Pore Diameter, nm	Ref.
1	0.053	494	24	This study
2	-	512	27	[45]
3	-	499–598	-	[19]
4	-	381–425	7.2–8.1	[46]
5	0.05–0.08	236–449	12–19	[47]

**Table 6:** Calculated values of components consumption during the solvent exchange process.

No.	2-Propanol Consumption, g	CO_2_ Consumption, g
1	765	800
2	530	813
3	446	856
4	424	783
5	368	704
6	217	243

## Data Availability

Data sharing not applicable.
